# Acute exercise alters skeletal muscle mitochondrial respiration and H_2_O_2_ emission in response to hyperinsulinemic-euglycemic clamp in middle-aged obese men

**DOI:** 10.1371/journal.pone.0188421

**Published:** 2017-11-21

**Authors:** Adam J. Trewin, Itamar Levinger, Lewan Parker, Christopher S. Shaw, Fabio R. Serpiello, Mitchell J. Anderson, Glenn K. McConell, David L. Hare, Nigel K. Stepto

**Affiliations:** 1 Institute of Sport, Exercise and Active Living (ISEAL), Victoria University, Melbourne, Australia; 2 Australian Institute for Musculoskeletal Science (AIMSS), Victoria University, St. Albans, Australia; 3 Institute for Physical Activity and Nutrition, School of Exercise and Nutrition Sciences, Deakin University, Geelong, Australia; 4 University of Melbourne, and Department of Cardiology, Austin Health, Melbourne, Australia; 5 Monash Centre for Health Research and Implementation (MCHRI), Monash University and Monash Health, Clayton, Australia; University of Birmingham, UNITED KINGDOM

## Abstract

Obesity, sedentary lifestyle and aging are associated with mitochondrial dysfunction and impaired insulin sensitivity. Acute exercise increases insulin sensitivity in skeletal muscle; however, whether mitochondria are involved in these processes remains unclear. The aim of this study was to investigate the effects of insulin stimulation at rest and after acute exercise on skeletal muscle mitochondrial respiratory function (*J*O_2_) and hydrogen peroxide emission (*J*H_2_O_2_), and the associations with insulin sensitivity in obese, sedentary men. Nine men (means ± SD: 57 ± 6 years; BMI 33 ± 5 kg.m^2^) underwent hyperinsulinemic-euglycemic clamps in two separate trials 1–3 weeks apart: one under resting conditions, and another 1 hour after high-intensity exercise (4x4 min cycling at 95% HR_peak_). Muscle biopsies were obtained at baseline, and pre/post clamp to measure *J*O_2_ with high-resolution respirometry and *J*H_2_O_2_ via Amplex UltraRed from permeabilized fibers. Post-exercise, both *J*O_2_ and *J*H_2_O_2_ during ADP stimulated state-3/OXPHOS respiration were lower compared to baseline (*P*<0.05), but not after subsequent insulin stimulation. *J*H_2_O_2_ was lower post-exercise and after subsequent insulin stimulation compared to insulin stimulation in the rest trial during succinate supported state-4/leak respiration (*P*<0.05). In contrast, *J*H_2_O_2_ increased during complex-I supported leak respiration with insulin after exercise compared with resting conditions (*P*<0.05). Resting insulin sensitivity and *J*H_2_O_2_ during complex-I leak respiration were positively correlated (*r* = 0.77, *P*<0.05). We conclude that in obese, older and sedentary men, acute exercise modifies skeletal muscle mitochondrial respiration and H_2_O_2_ emission responses to hyperinsulinemia in a respiratory state-specific manner, which may have implications for metabolic diseases involving insulin resistance.

## Introduction

More than one-third of the adult population worldwide are overweight or obese [1, 2]. Obesity increases the risk of developing insulin resistance, and this may be exacerbated by aging and sedentary lifestyle [3]. To counter this, regular exercise is a primary intervention for the prevention and management of metabolic diseases [4, 5]. The beneficial effects of exercise may occur in part by preventing or alleviating mitochondrial dysfunction which is thought to cause, or at least contribute to these pathophysiologic states [6–8]. Of note, even a single bout of exercise increases whole body insulin sensitivity, primarily in skeletal muscle, for up to 48-h post-exercise [9, 10]. However, whether skeletal muscle mitochondria are involved in mediating these effects after an acute bout of exercise currently remain unclear.

Exercise elicits disturbances to the skeletal muscle cellular environment, and this may transiently alter mitochondrial electron transport system (ETS) enzyme activity and mitochondrial membrane permeability in the hours post-exercise [11–13]. Mitochondrial ETS enzymes intrinsically generate reactive oxygen species (ROS), primarily in the form of superoxide which is rapidly dismutated to the membrane permeable hydrogen peroxide (H_2_O_2_). A number of sites within the ETS have been shown to generate ROS, and their rates of generation are largely determined by the mitochondrial respiratory state, which is linked to the bioenergetic demands of the cellular environment [14, 15]. During exercise mitochondrial ROS generation is decreased, but net muscle ROS generation is increased via non-mitochondrial sources such as NADPH and xanthine oxidases [16]. However, the sudden decrease in post-exercise energetic demand may have major impact on mitochondrial bioenergetics and therefore alter ROS generation, which could be also be amplified by numerous post-translational modifications to mitochondrial enzymes [15, 17]. Transient changes in ROS generation may have important retrograde signaling effects. For example, sustained or chronically elevated levels of ROS (i.e.: oxidative stress) have been implicated in insulin resistance via activation of stress activated protein kinases (SAPKs), which leads to insulin receptor substrate-1/2 (IRS1/2) serine phosphorylation, to negatively regulate phosphatidylinositol 3-kinase (PI3K) activity and its downstream signaling for GLUT-4 translocation and glucose uptake [18]. On the other hand, acute transient increases in ROS generation may oxidize and inhibit negative regulators of insulin signaling such as protein tyrosine phosphatase-1B (PTP1B) and phosphatase tensin homolog (PTEN), thus allowing PI3K phosphorylation [19–22].

In skeletal muscle mitochondria, nearly 100 mitochondrial proteins are known to be phosphorylated in response to a hyperinsulinemic-euglycemic clamp [23]. Consistent with the notion that mitochondrial dysfunction is associated with insulin resistance and likelihood of prediabetes [24], mitochondrial respiratory function has been shown to be elevated after a hyperinsulinemic-euglycemic clamp in insulin-sensitive individuals, but not in patients with type-2 diabetes [25–27]. Whether a similar mitochondrial-insensitivity to the insulin clamp is also characteristic of obese individuals is currently unclear. It was recently shown that obese women had unchanged *J*H_2_O_2_ after high fat feeding in the untrained state; yet following 12 weeks of exercise training, *J*H_2_O_2_ was elevated after the same meal, [28]. This suggests that greater mitochondrial sensitivity to nutrient intake and improved metabolic flexibility is an important component of exercise adaptation. Despite this, it remains unknown whether a single bout of exercise can similarly affect mitochondrial responses in an obese, older and sedentary population.

Therefore, the aims of this study were to test the hypothesis that in obese, older and sedentary individuals, mitochondrial respiration and H_2_O_2_ emission in permeabilized skeletal muscle fibers would be perturbed in a respiratory state-dependent manner in response to a hyperinsulinemic-euglycemic clamp to a greater extent when preceded by a single session of high intensity interval exercise (HIIE) compared to resting conditions. We also sought to explore the hypothesis that there would be correlation between rates of mitochondrial H_2_O_2_ emission and insulin sensitivity.

## Methods

### Participants

Nine obese and sedentary men (mean ± SD; age: 57.3 ± 6.5 years, body mass: 100.1 ± 12.1 kg, BMI: 32.7 ± 5.0 kg.m^-2^, $\dot{V}O_{2\mathrm{peak}}$: 21.4 ± 5.4 ml.kg^-1^.min^-1^) without diabetes (fasting glucose: 5.3 ± 0.8 mmol.L^-1^; HBa1c: 5.6 ± 0.2%) participated in this study. These participants were recruited as part of a larger study and as such, detailed participant characteristics and exclusion criteria are described elsewhere [29, 30]. Briefly, participants were excluded if they took medications known to affect insulin secretion and/or insulin sensitivity; had musculoskeletal or other conditions which prevented daily activity; or symptomatic or uncontrolled metabolic or cardiovascular disease. Each participant was given written and verbal explanations about the study before providing written informed consent as per the declaration of Helsinki. This study protocol was approved by Victoria University Human Research Ethics Committee.

### Experimental design

**Fig 1:**
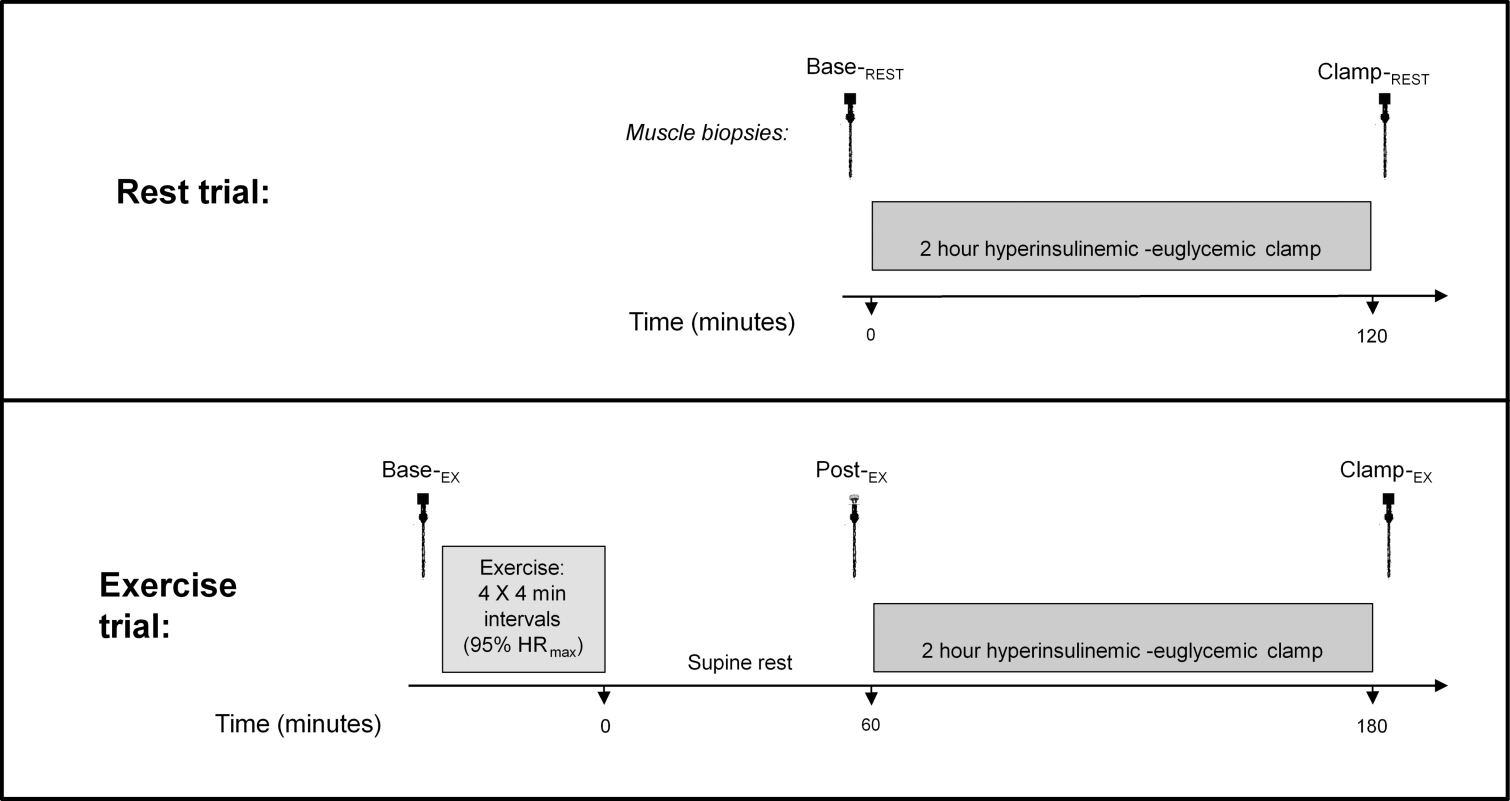
Overview of study design. Middle-aged obese men each performed two separate trials (rest and exercise) 1–3 weeks apart. Rest trial muscle biopsies were taken at baseline (Base-_REST_) and post 2 hour hyperinsulinemic-euglycemic insulin clamp (Clamp-_REST_); exercise trial biopsies were obtained at baseline (Base-_EX_), 1 hour post-exercise (Post-_EX_) and after a subsequent 2 hour hyperinsulinemic-euglycemic insulin clamp (Clamp-_EX_).

Participants visited the Victoria University Exercise Physiology laboratory on three separate occasions. In the first visit, participants underwent a screening and characterization session which included a graded exercise test and familiarization with the experimental procedures. At least one week following this, the first of the two experimental trials were conducted. As shown in Fig 1, the first experimental trial was a ‘rest trial’ (i.e. no exercise) and consisted of a 2 h hyperinsulinemic-euglycemic clamp (insulin clamp, see below). During this rest trial, muscle biopsies were obtained at baseline (Base-_REST_) and after the insulin clamp (Clamp-_REST_). The final visit was 1–3 weeks later for the ‘exercise trial’. In this, participants performed an acute HIIE exercise session (see below) then 1 hour later underwent another 2 h insulin clamp. In this exercise trial, biopsies were obtained at baseline (Base-_EX_), 1 h post exercise (Post-_EX_; which was immediately prior to the commencement of the insulin clamp), and then post insulin clamp (Clamp-_EX_). Prior to both trial days, participants performed an overnight fast, and abstained from physical activity for at least 72 h and alcohol and caffeine consumption for 24 h. Dietary information was provided and participants were asked to consume approximately 300 g of carbohydrate in the 24 h prior to the rest trial, which was recorded in a diet diary and replicated for the exercise trial.

### Graded exercise test

After pre-screening, participants underwent a sign and symptom-limited graded exercise test for characterization of aerobic fitness, as described elsewhere [31]. Oxygen uptake (VO_2_) was measured in 15 sec intervals by gas analysis (Medgraphics, Cardio2 and CPX/D System with Breezeex Software, 142090–001, Revia, MN, USA) calibrated with gases of known concentrations, and a 3 L volume Hans-Rudolph syringe prior to each test.

### High-intensity interval exercise protocol

At least a week after the graded exercise test, participants performed a bout of high-intensity intermittent exercise (HIIE) on a cycle ergometer (Corvial, Lode B.V., The Netherlands). The exercise consisted of 4 minutes warm-up at a workload corresponding to 50% of the peak heart rate (HR_peak_) obtained during the graded exercise test, followed by 4 x 4 min at 95% HR_peak_. HIIE bouts were separated by 2 min active recovery (cycling at a low intensity at 50–60% HR_peak_). All participants successfully completed the exercise session at the desired intensity.

### Hyperinsulinemic-euglycemic clamp

The insulin clamp was performed after an overnight fast, as previously described [29]. Briefly, insulin (Actrapid; Novo Nordisk, Denmark) was infused intravenously at 40 mU.m^-2^ per minute for 120 minutes leading to an elevated yet stable plasma insulin concentration from 10–120 min. Blood glucose concentration was assessed at 5 min intervals during the clamp (YSI 2300 STAT plus; YSI Inc., USA) and the glucose infusion rate (GIR; mg kg^-1^ min^-1^) was adjusted accordingly to meet a target blood glucose of 5 mmol L^-1^. Insulin sensitivity was determined as the mean GIR per unit plasma insulin (mIU ml^-1^) during the last 30 min of the clamp (M/I index) [32].

### Muscle biopsy procedure

Overall, five muscle biopsies were obtained from each participant (Fig 1). Local anesthetic (1% Xylocaine, AstraZeneca, Australia) was injected into skin, subcutaneous tissue and fascia overlying the *vastus lateralis* muscle. After small incisions were made into the skin and fascia (one per biopsy), muscle was excised using a Borgström needle with suction [29, 30, 33]. Each biopsy was taken from a separate incision ~1 cm proximal from the previous biopsy. Samples of approximately 50–100 mg were obtained and aliquoted for separate analysis including ~10 mg which was immediately placed in an ice-cold preservation medium (BIOPS, see below) for mitochondrial respiration analysis on the same day, while the remaining portion was blotted to remove blood and rapidly frozen in liquid nitrogen and stored at -80°C for later analysis.

### Muscle fiber preparation for mitochondrial respiratory function analysis

The ~10 mg aliquot of muscle was placed in ice-cold preserving solution (BIOPS; in mM: 7.23 K_2_EGTA, 2.77 CaK_2_EGTA, 5.77 Na_2_ATP, 6.56 MgCl_2_-6H_2_O, 20 taurine, 15 phosphocreatine, 20 imidazole, 0.5 dithiothreitol, 50 K^+^-MES; pH 7.1) until analysis, typically within 3 hours after sampling [34–36]. Using a dissecting microscope, muscle fibers were mechanically separated using fine-point forceps for no more than 3 min while submerged in ice-cold BIOPS. Separated fibers were permeabilized (saponin 50 μg/mL in BIOPS) for 30 min with agitation followed by 3 x 5 min washes in ice-cold respiration buffer (MiR05, see below). Fiber bundles of approximately 2–3 mg were blotted on filter paper for 5 s, then exact sample mass (wet-weight) was recorded using a microbalance (Cubis MSE3.6P-0TR-DM, Sartorius, Goettingen, Germany).

### Mitochondrial respiration and hydrogen peroxide emission assay

Mitochondrial oxygen flux (*J*O_2_) from permeabilized muscle fibers was determined using high resolution respirometry at 37°C, high oxygen concentration (300–450 nmol.ml^-1^; to avoid oxygen diffusion limitation) and continuous stirring (Oxygraph O2k, Oroboros Instruments, Innsbruck, Austria), and mitochondrial hydrogen peroxide emission (*J*H_2_O_2_) was measured simultaneously via fluorimetry (O2k-Fluorescence LED-2 Module; Oroboros Instruments, Innsbruck, Austria) as previously described [36–39]. Briefly, permeabilized fiber bundles were analysed in duplicate, in chambers containing MiR05 respiration buffer (in mM: 0.5 EGTA, 10 KH_2_PO_4_, 3 MgCl_2_-6H_2_O, 60 lactobionic acid, 20 taurine, 20 HEPES, 110 D-sucrose, 1 mg/mL bovine serum albumin; pH 7.1). Amplex UltraRed (25 μM; Molecular Probes, Invitrogen), horseradish peroxidase (5 U/mL) and superoxide dismutase (SOD; 5 U/mL) were added for simultaneous fluorimetric measurement of H_2_O_2_ at 525/590 nm excitation/emission wavelengths, calibrated with known amounts of H_2_O_2_ as described previously [36, 37, 39]. A range of respiratory states were induced using a substrate, uncoupler, and inhibitor titration (SUIT) protocol, added sequentially as follows: malate (2 mM), pyruvate (5 mM) and octanoylcarnitine (0.02 mM) to assess complex I + electron transfer flavoprotein (ETF) supported state-4 leak respiration (*LEAK*_CI+ETF_); succinate (10 mM) was then added for convergent complex II electron input during leak respiration (*LEAK*_CI+II+ETF_); ADP (1 & 5 mM) was then added to induce state-3 oxidative phosphorylation (*OXPHOS*); cytochrome-c (10 μM) was added to confirm outer mitochondrial membrane integrity; stepwise 0.05 μM titrations of carbonyl cyanide *p*-trifloromethoxyphenylhydrazone (FCCP) were then added to uncouple the inner mitochondrial membrane to assess electron transfer system capacity (*ETS*_CI+II+ETF_). The complex I specific inhibitor rotenone (0.5 μM) was subsequently added to assess electron transfer from complex II (*ETS*_CII+ETF_). Finally, antimycin-A (2.5 μM) was added to inhibit complex III to determine residual oxygen flux rates which were subtracted from all prior *J*O_2_ measures.

### Muscle protein extraction and western blotting

Abundance of specific proteins in muscle samples were determined without centrifugation (i.e. all cellular fractions present) using methods described previously [40]. Specifically, frozen muscle was cut into approximately 20 x 20 μm sections (Cryostat HM550, Thermo Scientific, Australia), and dissolved in 200 μL homogenizing buffer (0.125 M Tris-HCl, 4% SDS, 10% Glycerol, 10 mM EGTA, 0.1 M DTT, with 0.1 μL.mL^-1^ of protease and phosphatase inhibitor cocktail [#P8340 and #P5726, Sigma Aldrich, Castle Hill, NSW, Australia]), vortexed and freeze-thawed. Protein concentration was then determined using a commercially available assay (Red 660, G-Biosciences, Astral Scientific, Gymea NSW, Australia). Samples were diluted to equivalent concentrations (1 μg.μL^-1^) in homogenizing buffer and bromophenol blue added (1% v/v) before heating at 95°C for 5 min. Samples were loaded onto 26 well, stain-free, precast 4–20% gradient gels (Criterion™ TGX Stain-Free™ Precast, BioRad, Gladesville NSW, Australia) at a concentration of 6–8 μg protein per lane. Molecular weight marker (PageRuler® Plus, Thermo Scientific, Australia) and a five-point standard curve was also loaded on each gel using a pooled sample allowing quantification of blot intensities within and between gels via linear regression. Optimal protein loading was determined to ensure that subsequent blot intensities were within the linear range of the standard curve [40]. After separation by SDS PAGE, stain-free gels were activated by UV light (ChemiDoc™ MP, BioRad, Gladesville NSW, Australia) and imaged to visualize the total protein of each lane then the proteins were transferred to PVDF membranes (Trans-Blot® Turbo™, BioRad, Gladesville NSW, Australia). Membranes were then blocked in 20 mM Tris, 150 mM NaCl, and 0.1% Tween 20 (TBST) containing 5% nonfat milk for 1 h at room temperature, washed, then incubated with primary antibody overnight at 4°C. Membranes were incubated with the following primary antibodies diluted 1:1000 in TBST containing 5% BSA and 0.1% sodium azide: mitochondrial complexes I-V cocktail (MitoSciences #MS601), PRX pathway cocktail (Abcam #184868), and UCP3 (Abcam #10985). Membranes were washed with TBST, then probed with appropriate horseradish peroxidase-conjugated secondary antibody (PerkinElmer, Australia) diluted 1:50,000 in 5% non-fat milk/TBST for 1 hour at RT. Protein-antibody-HRP conjugates were visualized by chemiluminescence using ECL detection (SuperSignal® West Femto, Thermo Scientific, Australia), imaged (ChemiDoc™ MP, BioRad, Australia) and then analysed (ImageLab v5.1, BioRad, Australia). Specifically, immunoblots for proteins of interest and associated stain-free total protein loading were quantified relative to their respective standard curves, and these values were then used to report protein of interest relative to total protein [40]. In the absence of stain-free loading control data for mitochondrial complexes due to equipment fault. Blot intensities derived from the mitochondrial cocktail antibody served as their own loading control since these were imaged on the same membrane. This was achieved by first normalizing each samples blot intensity to a pooled sample loaded on all membranes, then, were expressed relative to complex-V within the same image, and complex-V was expressed relative to complex-III.

### Citrate synthase activity assay

Citrate synthase activity was performed as per Srere [41] modified for a 96 well plate format [34, 35]. Muscle was mechanically homogenized 1:20 w/v in buffer (0.175 M KCl; 2 mM EDTA; pH 7.4) then freeze-thawed. Sample (10 μL) was added to 190 μL of working solution (final concentrations in mM: 72.5 tris, 0.1 DTNB, 0.45 acetyl co-A, 0.25% v/v Triton X-100) followed by 10 μL of oxaloacetic acid (0.5 mM) to initiate the reaction. Citrate synthase (CS) activity at 30°C was determined by the change in absorbance at 412 nm over 4 min in a spectrophotometer (x-Mark, Bio-Rad laboratories, USA).

### Reduced and oxidized glutathione assay

Reduced (GSH) and oxidized (GSSG) muscle glutathione content was determined using a commercially available kit (Bioxytech GSH/GSSG-412, Oxis Health Products, Portland, OR, USA) described previously [42]. Briefly, freeze dried muscle was dissected free of connective tissue, divided into two aliquots then homogenized in 80 μl.mg^-1^ (dry weight) ice-cold 5% metaphosphoric acid, one aliquot containing the reduced-glutathione scavenger 1-methyl-2-vinyl-pyridinium trifluoromethane sulphonate (10% v/v) for GSSG, and the other aliquot without for GSH. Homogenate was centrifuged at 23,000 *g* for 15 min at 4°C. Samples, standards and blanks (50 μl) were added to a 96 well plate in triplicate, followed by 50 μl each of chromogen, glutathione reductase and just prior to measurement, NADPH. Change in absorbance at 412 nm due to the reduction of DTNB was measured for 4 min in a spectrophotometer (xMark; Bio-Rad Laboratories, USA).

### Statistical analysis

Owing to the study design with two time points in the rest trial, and three in the exercise trial, between-trial effects at baseline and post-insulin stimulation were analysed by two-way (intervention x time point) ANOVA with repeated measures; whereas effects within the exercise trial were assessed by repeated measures one-way ANOVA with Fisher’s LSD post-hoc tests using statistical software (IBM SPSS statistics version 22). Significance was accepted at *P*<0.05. Associations between insulin sensitivity and *J*H_2_O_2_ after the rest and post-exercise insulin clamp were determined using Pearson’s correlation coefficient. All data are presented as mean ± SD for *n* = 9 unless otherwise stated.

## Results

### Mitochondrial respiration

**Fig 2:**
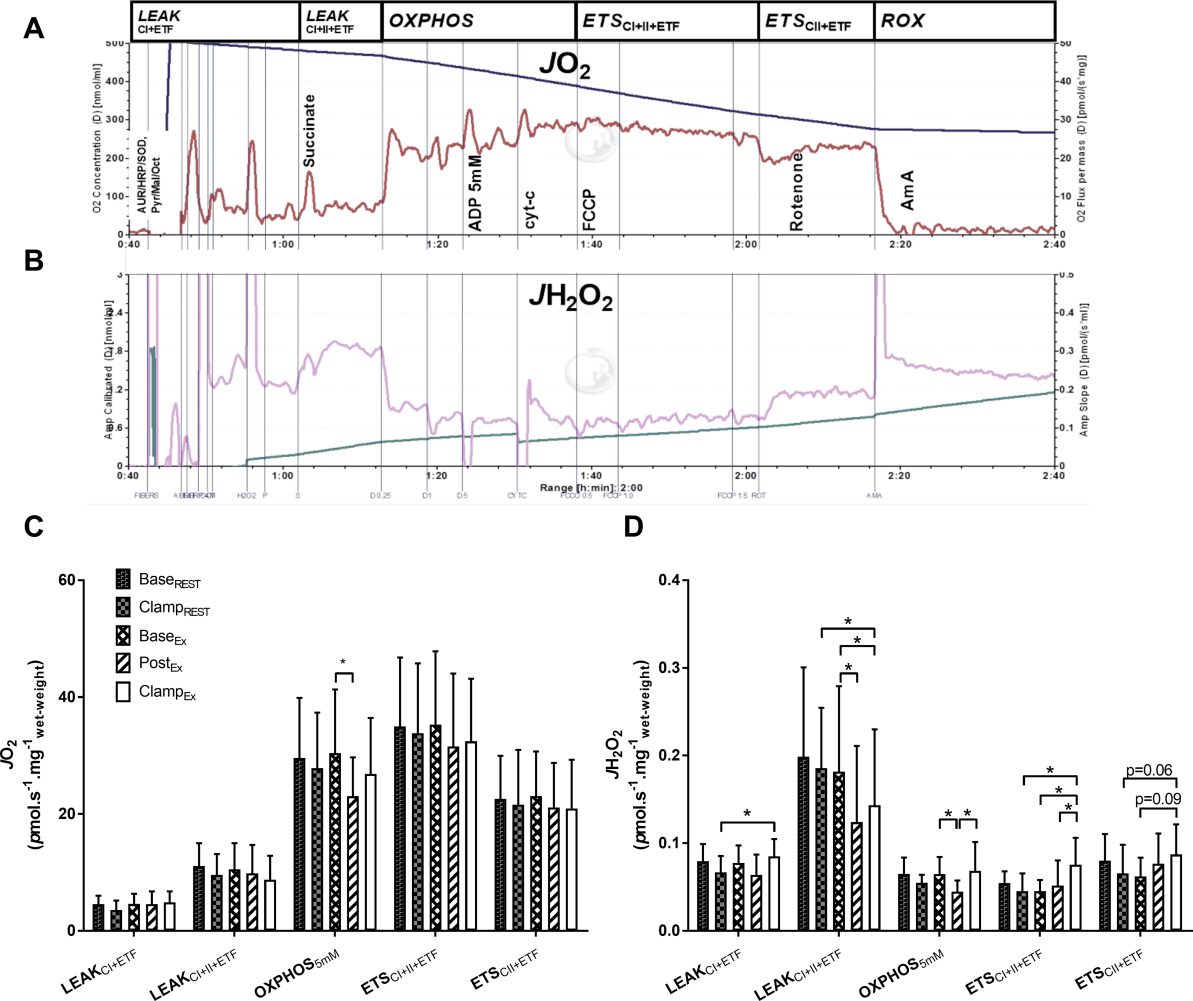
Oxygen flux (*J*O_2_) and mitochondrial hydrogen peroxide emission (*J*H_2_O_2_) from permeabilized skeletal muscle fibers during various mitochondrial respiratory states, pre- and post-exercise and/or insulin clamp. Representative traces of *J*O_2_ (A) and *J*H_2_O_2_ (B) are shown from one subject, and overall data are quantified (C and D). See *Methods* for abbreviations and details of the SUIT protocol. Values are mean ± SD for *n* = 9. *P<0.05 significantly different.

**LEAK respiration:** Throughout the SUIT protocol (Fig 2A), there were no significant effects of exercise and/or insulin on *J*O_2_ during *LEAK*_CI+ETF_ respiration state; however, in the presence of complex II substrate succinate (*LEAK*_CI+II+ETF_), *J*O_2_ trended lower in the rest trial after insulin compared to baseline (*P* = 0.09; Fig 2C).

**OXPHOS respiration:** There was no effect of insulin on *J*O_2_ under maximal (5 mM ADP) *OXPHOS* respiratory state in the rest trial. Compared to baseline, *J*O_2_ during *OXPHOS* respiration was lower 1-hr post exercise (*P*<0.01; Fig 2C), which then returned to baseline levels with subsequent insulin stimulation. Titration of cytochrome-C increased *OXPHOS* respiration *J*O_2_ by an average of 7.6% across the study. Respiratory control ratios were not significantly affected for *OXPHOS* / *LEAK*_CI+ETF_ (mean±SD): Base-_REST_ 7.6 ± 5.1; Clamp-_REST_ 8.9 ± 4.1; Base-_EX_ 8.1 ± 5.4; Post-_EX_ 5.7 ± 2.1; Clamp-_EX_ 6.2 ± 2.8; or for *OXPHOS* / *LEAK*_CI+II+ETF_: Base-_REST_ 2.7 ± 0.7; Clamp-_REST_ 3.0 ± 0.9; Base-_EX_ 3.0 ± 0.7; Post-_EX_ 2.6 ± 0.7; Clamp-_EX_ 3.4 ± 1.4.

**ETS uncoupled respiration:** There were no significant effects of insulin and/or exercise on *J*O_2_ during uncoupled respiration when supported by convergent complex I, II and ETF electron input (*ETS*_CI+II+ETF_) or with electron input in the absence of complex I after rotenone inhibition (*ETS*_CII+ETF_; Fig 2C).

### Mitochondrial H_2_O_2_ emission

**LEAK respiration:** Throughout the SUIT protocol (Fig 2B), mitochondrial hydrogen peroxide emission (*J*H_2_O_2_) during *LEAK*_CI+ETF_ respiration was greater after insulin in the exercise trial compared to the resting trial (*P*<0.01; Fig 2D). During *LEAK*_CI+II+ETF_ respiration (with added succinate), *J*H_2_O_2_ was lower at both Post-_EX_ (*P =* 0.01; Fig 2D) and Clamp-_EX_ (*P =* 0.05; Fig 2D) compared to Base-_EX_. In addition, during *LEAK*_CI+II+ETF_, there was lower *J*H_2_O_2_ with insulin in the exercise trial compared to the resting trial (*P =* 0.02; Fig 2D).

**OXPHOS respiration:** Under 5 mM ADP stimulated *OXPHOS* respiration, *J*H_2_O_2_ was lower after exercise compared to baseline (*P =* 0.03; Fig 2D) and also compared to subsequent insulin (*P =* 0.02; Fig 2D). There was no effect of the subsequent cytochrome-c titration on *J*H_2_O_2_, as determined from combined data of all nine participants across five conditions (pre *v*.*s*. post cytochrome-c titration *J*H_2_O_2_: 0.058 ± 0.020 *v*.*s*. 0.058 ± 0.029 *p*mol.s^-1^.mg^-1^ tissue wet weight, *P* = 0.95, *n* = 45).

**ETS uncoupled respiration:** In the uncoupled respiration state (*ETS*_CI+II+ETF_) in the exercise trial, *J*H_2_O_2_ was greater with insulin compared to both baseline (*P* = 0.02) and post-exercise (*P* = 0.04; Fig 2D). Furthermore, *J*H_2_O_2_ was greater in this respiratory state with insulin in the exercise trial compared to the resting trial (*P* = 0.01; Fig 2D). In the presence of rotenone to inhibit complex-I electron input (*ETS*_CII+ETF_), in the exercise trial there was a trend for elevated *J*H_2_O_2_ after insulin stimulation compared with both baseline (*P* = 0.09) and with insulin in the rest trial (*P* = 0.06; Fig 2D).

### Glucose infusion rate

GIR was 26% greater after exercise compared with the resting trial (4.21 ± 2.41 *v*.*s*. 3.33 ± 2.16 mg.kg^-1^.min^-1^; *P* = 0.03; S1A Fig). Plasma hyperinsulinemia induced by the clamp was stable in the last 30 min of the clamp and equivalent between trials (Clamp-_REST_: 73.6 ± 24.5 mIU L^-1^ and Clamp-_EX_: 67.1 ± 20.5 mIU L^-1^; *P* = 0.37; S1B Fig). GIR per unit plasma insulin was 27% greater post-exercise compared to rest (6.26 ± 3.6 *v*.*s*. 4.95 ± 3.77 M/I index; *P* = 0.04; S1C Fig). Blood glucose concentration was well maintained at ~5 mmol.L^-1^ in the final 30 minutes of the clamps (coefficient of variation: 2.1%, S1D Fig).

### Correlations between insulin sensitivity and mitochondrial H_2_O_2_

**Fig 3:**
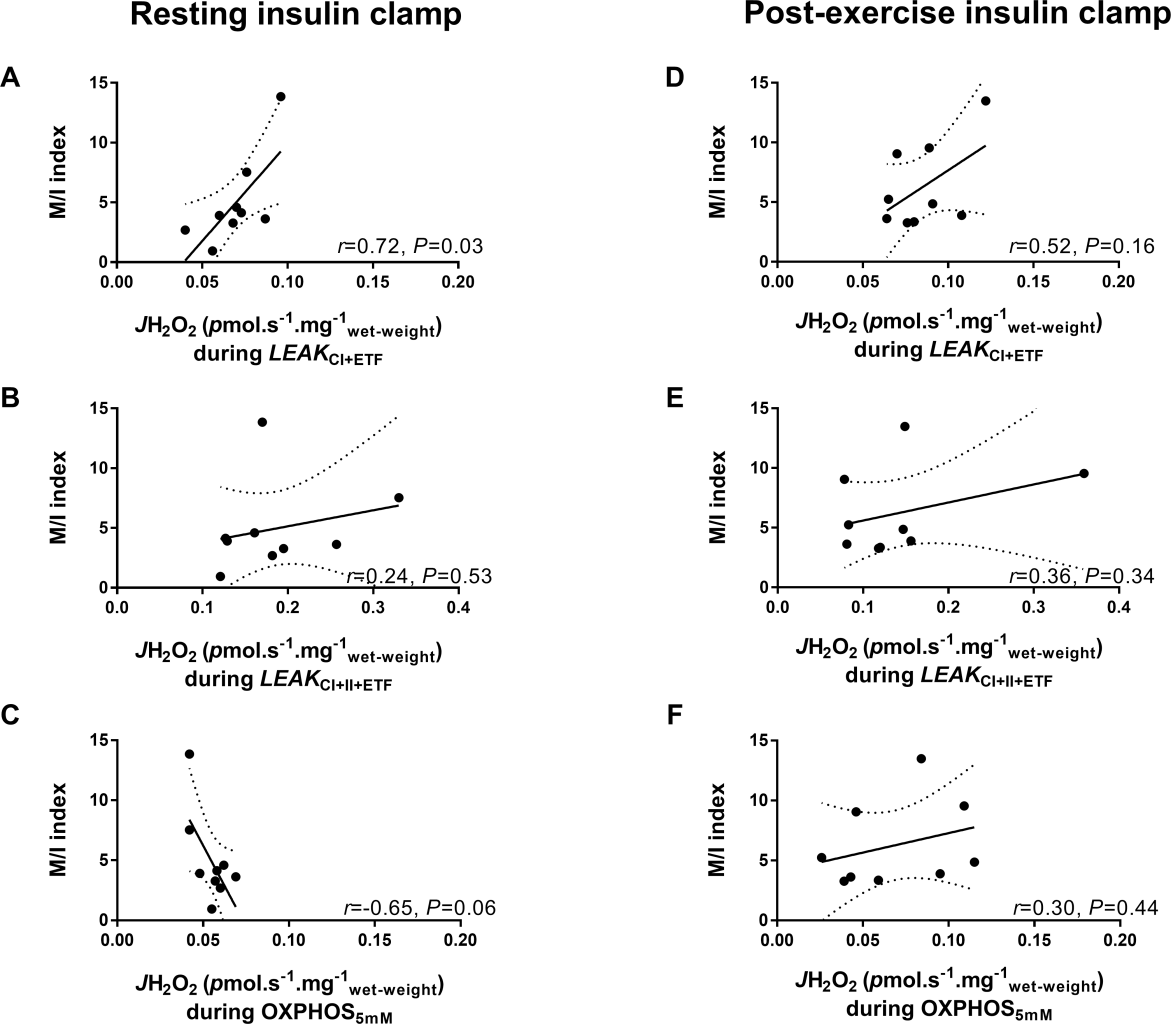
Correlations between insulin sensitivity (M/I index) and mitochondrial H_2_O_2_ emission. Comparisons are made after the resting insulin clamp (A-D) and the post-exercise insulin clamp (E-G) under specific respiratory states: *LEAK*_*CI+ETF*_ (A & D), *LEAK*_*CI+II+ETF*_ (B & E), and *OXPHOS*_*5mM*_ (C & F). M/I index is insulin sensitivity: glucose infused (mg.kg^-1^.min^-1^) per unit plasma insulin (mIU ml^-1^). Dotted lines represent 95% confidence bands of the best-fit line for *n* = 9.

There was a significant correlation between whole body insulin sensitivity after the resting clamp and *J*H_2_O_2_ during *LEAK*_CI+ETF_ (*r* = 0.72; *P* = 0.03; Fig 3A), however, this association was not significant post-exercise (*r* = 0.52; *P* = 0.16; Fig 3D). There was a trend for a negative correlation during *OXPHOS*_5mM_ at rest (r = -0.65, *P* = 0.06; Fig 3C). There were no correlations between *J*H_2_O_2_ and insulin sensitivity during *LEAK*_CI+II+ETF_ (Fig 3B and 3E), or either uncoupled *ETS* respiratory states (S1 File).

### Muscle glutathione content

**Fig 4:**
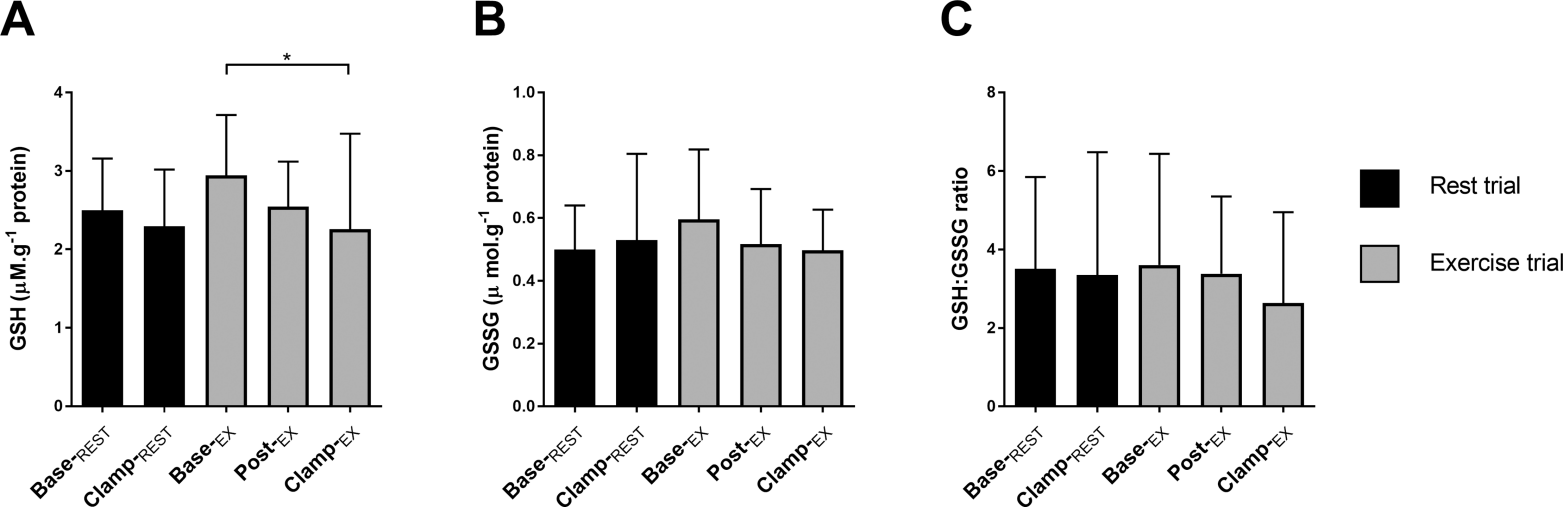
Muscle glutathione status. Reduced glutathione (A, GSH), oxidized glutathione (B, GSSG) content was normalized to total protein of whole muscle homogenate and expressed as a ratio (C). Values are mean ± SD for *n* = 9. *P<0.05 significantly different.

Reduced-glutathione (GSH) was lower after insulin compared with baseline in the exercise trial (*P =* 0.04; Fig 4A). There was no significant effect of insulin or exercise on oxidized-glutathione (GSSG; Fig 4B), or on the ratio of GSH to GSSG (Fig 4C).

### Mitochondrial protein abundance and citrate synthase activity

**Fig 5:**
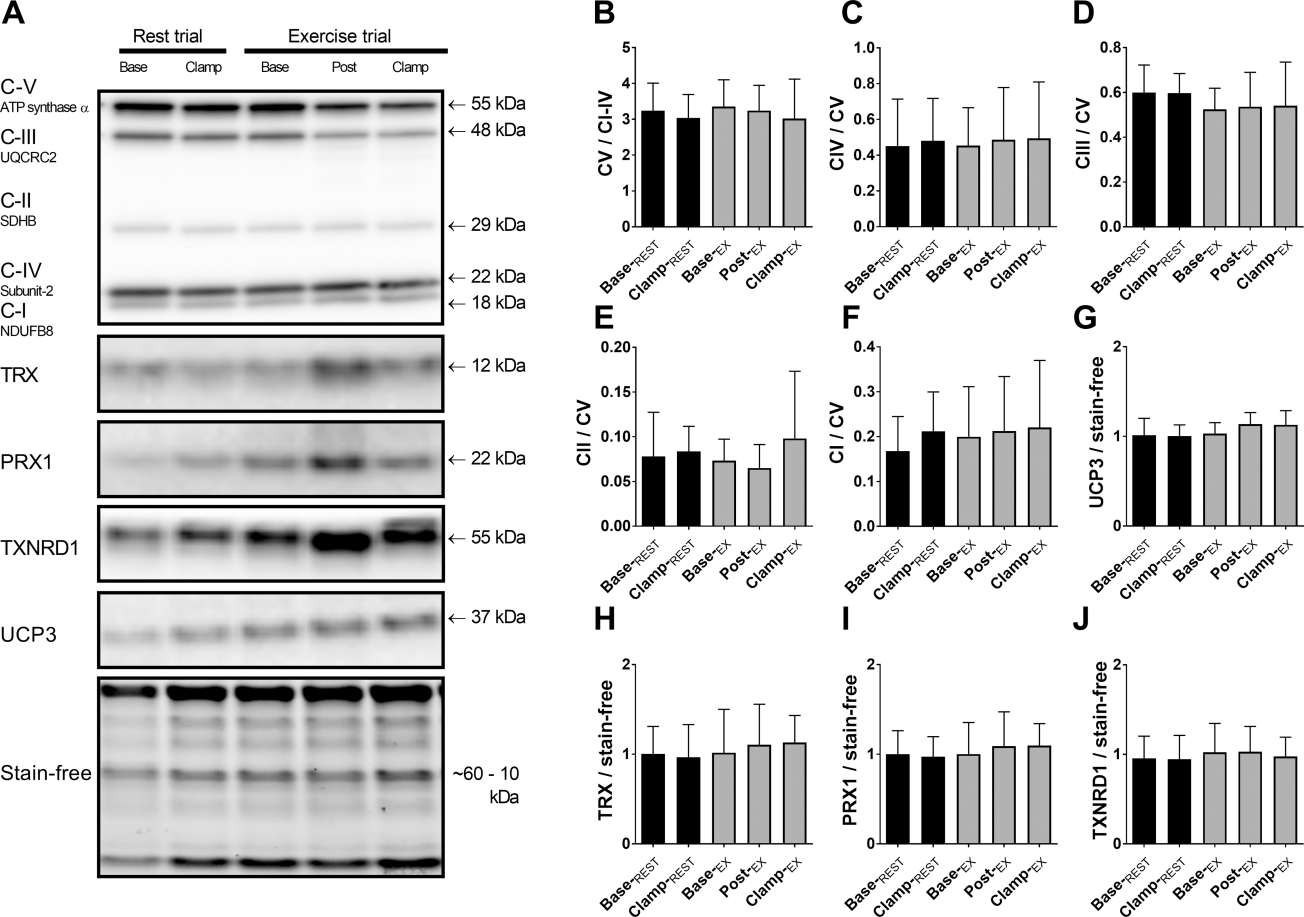
Mitochondrial and antioxidant proteins from whole muscle homogenate. Representative blots depict typical immunoblots from one subject (A). Immunoblots of mitochondrial complex V (CV) are expressed relative to average intensities of CI to IV of the same lane (B); complexes IV, III, II and I are normalized to CV of each lane (C-F). Uncoupling protein-3 (G), antioxidant enzymes TRX (thioredoxin, H), PRX1 (peroxiredoxin-1, I), and TXNRD1 (thioredoxin reductase-1, J) normalized to whole sample protein determined from stain-free image. Values are mean ± SD for *n* = 9.

Mitochondrial ETS proteins were assessed to confirm that acute changes in mitochondrial respiration were independent of changes in their abundance. There were no significant changes in any of the measured ETS subunits, or uncoupling protein-3 (Fig 5B–5G). Citrate synthase enzyme activity, an additional marker of mitochondrial abundance, was unchanged with exercise or insulin (means±SD: Base-_REST_: 4.6 ± 2.5; Clamp-_REST_: 5.0 ± 1.7; Base-_EX_: 5.3 ± 3.0; Post-_EX_: 4.3 ± 3.4; Clamp-_EX_: 4.6 ± 2.3 μmol.min^-1^.g-protein^-1^; *n*.*s*.).

### Muscle endogenous antioxidant protein abundance

To investigate possible reasons for the transient shifts in mitochondrial H_2_O_2_ emission observed, muscle antioxidant enzyme abundance were measured at corresponding time points. There was no significant change in muscle protein content of thioredoxin (TRX), peroxiredoxin-1 (PRX1) or thrioredoxin reductase-1 (TXNRD1) at any time points (Fig 5H–5J).

## Discussion

The main findings of this study were that following acute exercise, but not under resting conditions, mitochondrial H_2_O_2_ emission was altered in a respiratory state-specific manner in response to hyperinsulinemic-euglycemic clamp in obese, middle-aged and sedentary men. In addition, there was a post-exercise decrease in rates of ADP stimulated mitochondrial respiration and H_2_O_2_ emission prior to insulin stimulation. There was also a significant correlation between whole body insulin sensitivity and skeletal muscle mitochondrial H_2_O_2_ emission during *LEAK*_CI+ETF_ respiration at rest; however, in contrast to our hypothesis, this relationship was not observed post-exercise.

Our finding that mitochondrial *J*H_2_O_2_ was lower one hour post-exercise in the physiologically relevant *OXPHOS*/state-3 respiration, is important since oxidative stress (i.e. chronically elevated ROS levels) is involved in the pathophysiology of various chronic diseases [43]. Indeed, exercise training is known to attenuate chronic oxidative stress [44, 45], and our data suggest that this improvement may begin even with a single bout of exercise. It should be noted that although ROS formation is highly dependent on membrane potential and the subsequent redox status of electron carriers in the ETS, some ROS formation is expected even under low membrane potential respiratory states (i.e. *OXPHOS* and uncoupled *ETS*) [14, 15].

To the best of our knowledge, only one study has measured H_2_O_2_ emission permeabilized skeletal muscle mitochondrial following acute exercise [46]. While that study was performed with rodents, their data indicated a trend for pyruvate+malate+succinate supported H_2_O_2_ emission to be lower in white gastrocnemius muscle 18 hours post exercise [46], and this is in line with the decreased post-exercise *LEAK*_CI+II+ETF_ *J*H_2_O_2_ (supported by malate+pyruvate+octanoylcarnitine and succinate) in the present study. The decrease in post-exercise/insulin clamp *J*H_2_O_2_ and *J*O_2_ during succinate-driven state-4/leak respiration is also consistent with a recent finding that ROS generation from complex I (via reverse electron flow) is inhibitory to complex II function [47]. Specifically, in that study, small molecule inhibitors of the complex-I ubiquinone binding site were shown to attenuate superoxide/H_2_O_2_ generation, which attenuated pathophysiological responses [47]. Taken together with our findings of decreased post-exercise H_2_O_2_ emissions during *LEAK*_CI+II+ETF_ respiration, acute exercise may elicit similar beneficial effects in obese, middle-aged and sedentary men via similar mechanisms which warrants further investigation.

On the other hand, we observed elevated mitochondrial H_2_O_2_ in response to the post-exercise insulin clamp during *LEAK*_CI+ETF_ and uncoupled *ETS*_CI+II+ETF_ respiratory states (Fig 2D) which may be pertinent, since ROS generation in specific spatial and temporal patterns at physiological levels is known to have an important role in cell signal transduction [21, 48]. Our findings are consistent with a recent study in obese women, which demonstrated a lack of response of *J*H_2_O_2_ after a high fat meal in the untrained state, yet after 12 weeks of exercise training there was an acute increase in *J*H_2_O_2_ in response to the same meal [28]. In line with this, in the present study individuals with greater insulin sensitivity at rest tended to have lower mitochondrial H_2_O_2_ emission during the ADP stimulated oxidative phosphorylation respiration state, but significantly greater mitochondrial H_2_O_2_ emission during conditions of high membrane potential (*LEAK*_CI+ETF_). This may allow a greater dynamic range of ROS generation, which could be important for signal transduction purposes, such as post-exercise insulin signaling. Notably, AS160 an essential regulatory protein in the distal insulin signaling pathway, has been demonstrated to be sensitive to H_2_O_2_ in rodents [20] while infusion of the antioxidant N-acetylcysteine was shown to attenuate post-exercise whole-body insulin sensitivity in young healthy humans [42]. Exercise is known to an additive effect on insulin induced phosphorylation of AS160 which is a key mediator of the post-exercise enhancement of skeletal muscle insulin sensitivity [49–51]. Indeed, in the present study, phosphorylation of both Ser588 and Ser318 of AS160, were greater after the post-exercise insulin clamp compared to the resting insulin clamp [30]. Despite this, in the present study, we were unable to detect significant correlations between H_2_O_2_ emission and post-exercise insulin sensitivity in our obese subjects. Further investigation is required to better understand these potential molecular interactions.

Several potential mechanisms may explain the altered H_2_O_2_ emission with post-exercise insulin clamp observed in the present study. The lower muscle GSH content following the post-exercise insulin clamp (Fig 4) suggests that some perturbation to muscle redox homeostasis occurred. Even small changes in concentration of the reduced glutathione (GSH), a key thiol antioxidant *in vivo* and substrate for glutathione peroxidase, has been shown to have significant effects on redox homeostasis [52]. Therefore, decreased GSH may permit the elevated rates of mitochondrial H_2_O_2_ emission during *LEAK*_CI+ETF_ and *ETS*_CI+II+ETF_. The lack of a corresponding increase in post-exercise oxidized glutathione (GSSG) reported in young healthy adults [42] could be related to the specific population studied presently, although the minor difference in baseline values between trials should also be considered when interpreting these data. In the present study, six out of the nice participants showed a post-exercise increase the total abundance of thioredoxin (Trx) and peroxiredoxin-1 (Prx1) and thioredoxin reductase (TXNRD1), but overall this was not significantly changed with exercise and/or insulin. These proteins play a key role in the modulation of low concentrations of ROS, due to their low *K*_M_ for H_2_O_2_ [52], and therefore in spatio-temporal redox-signaling [53, 54]. Although our data are inconclusive, some of the change in mitochondrial *J*H_2_O_2_ may be partly explained due to acute changes in the total antioxidant capacity conferred by the Trx/Prx system. In addition, it is possible that various post-translational protein modifications not measured in the present study could acutely alter the post-exercise enzymatic activity of these and other mitochondrial antioxidant enzymes such as SOD1 and GPx1 [53]. Taken together, these findings may suggest that even in an aging and obese population there remains a ‘functional reserve’ in antioxidant capacity to allow for spatio-temporal redox-mediated cell signaling without inducing a state of oxidative stress [54].

Our findings of a transient decrease in *J*O_2_ during *OXPHOS* after exercise are in line with previous studies which report impaired post-exercise respiratory complex enzyme function [12, 55], and decreased *OXPHOS J*O_2_ in permeabilized muscle fibers following high intensity exercise in horses [13, 46, 56]. In permeabilized skeletal muscle mitochondria of young healthy humans, Tonkonogi *et al*. [57] have reported that state-3 (i.e. ‘*OXPHOS’*) respiratory rates are increased immediately and 2 hours post-exercise, while Perry *et al*. [58] reported no change immediately or 3 hours post exercise on maximal *OXPHOS J*O_2_. However, direct comparisons to their findings are difficult to make with our subjects due to the much lower *OXPHOS J*O_2_ rates and respiratory control ratios, as expected [59], and in these previous studies state-3 respiratory measures were supported by pyruvate+malate (complex-I) alone, whereas in the present study there was convergent substrate input from pyruvate, malate, octanoylcarnitine as well as succinate (complex-II). Interestingly, in our obese, older and sedentary subjects, there was no increase of *J*O_2_ in response to insulin, which appears to be consistent with what has been previously demonstrated in patients with type-2 diabetes when compared with young, healthy individuals [26, 60, 61]. The lack of response in *J*O_2_ to insulin in our subjects with obesity could therefore be interpreted as the early signs of impaired metabolic flexibility which is observed in type-2 diabetes [62]. An alternative explanation is that the aforementioned studies co-infused amino acids to avoid low plasma amino acid concentrations which can occur during longer durations (4–8 hours) of insulin infusion, which may have provided sufficient time and stimulus for the synthesis of new mitochondrial proteins. Indeed, more recent studies have reported that in the absence of amino acid co-infusion, insulin did not appear to increase mitochondrial ATP synthesis rates, even in young healthy individuals [25, 63].

There are some potential limitations to the present study. The small sample size, while typical of similar invasive human studies, may preclude the possibility of detecting potentially small effects between insulin sensitivity and mitochondrial H_2_O_2_ emission. Despite this, post hoc power analysis demonstrated that the reported reduction (~30%) of *J*H_2_O_2_ during *OXPHOS* after exercise with *n* = 9 and *α* = 0.05 that the study was adequately powered (93%). It is acknowledged that the lack of a non-obese control group limits the ability to specifically ascribe the present findings to that of a pathophysiological response. The present study design would be strengthened with an additional exercise session with a 3 hour post exercise muscle sample in the absence of hyperinsulinemia, to allow for a more robust comparison of the specific effects of insulin on transient changes in mitochondrial function after exercise. Also, it cannot be excluded that a longer rest period between the cessation of exercise and the start of the insulin clamp may be required to measure the effects of insulin-stimulated glucose uptake independent of any residual effects of contraction-mediated glucose uptake [10]. The study design could potentially be influenced by the different number of muscle biopsies obtained in each trial since repeated muscle biopsies can impact glycogen resynthesis [64, 65]. However, this seems unlikely as these effects were observed over a longer period of time (48 h) and another study reported no effect of repeated biopsies within a shorter 5–6 hour window [66]. Furthermore, it has been shown that repeated biopsies 1 hour apart from adjacent sites of the same muscle had no effect on ERK1/2 phosphorylation [67]. Cytochrome-c added to the mitochondrial respiratory assay is used to assess potential damage to the outer mitochondrial membrane during sample preparation and it should be acknowledged that the redox-active heme group in cytochrome-c may scavenge unpaired electrons and thereby potentially influence the subsequent detection of H_2_O_2_. Therefore, although in the present study cytochrome-c was added only after *OXPHOS* respiration states and at the same concentration in all experiments, our *J*H_2_O_2_ data in the presence of cytochrome-c should nonetheless be interpreted accordingly. The present method for measuring *J*O_2_ and *J*H_2_O_2_ has been well characterized previously [37–39], although some caution should be used when interpreting these findings in the context of *in vivo* physiology due to the use of hyperoxygenation (to avoid oxygen diffusion limitations) and saturating substrate concentrations in the permeabilized muscle fiber mitochondrial respiration assay. It is also acknowledged, due to practical limitations, we did not assess malalate+pyruvate and octanoylcarnitine supported *J*O_2_ and *J*H_2_O_2_ separately. These limitations should therefore be considered in future investigations.

In conclusion, this study provides novel evidence that a single bout of aerobic exercise acutely modifies skeletal muscle mitochondrial respiration and H_2_O_2_ emission and responses to insulin stimulation in obese, middle-aged and sedentary males, and this may have implications for metabolic diseases featuring insulin resistance.

## Supporting information

S1 Fig Hyperinsulinemic-euglycemic clamps under resting conditions and 1 h post-exercise. (A) Mean glucose infusion rate, (B) plasma insulin concentration and (C) M/I index data plotted for individual subjects for the final 30 min of each clamp. (D) Blood glucose concentrations for the final 30 min of the clamps, data are mean ± SD for *n* = 9.
(TIF)

S1 File Anonymized dataset. (XLSX)
